# Tuberculosis Treatment Managed by Providers outside the Public Health Department: Lessons for the Affordable Care Act

**DOI:** 10.1371/journal.pone.0110645

**Published:** 2014-10-23

**Authors:** Melissa Ehman, Jennifer Flood, Pennan M. Barry

**Affiliations:** 1 Tuberculosis Control Branch, Division of Communicable Disease Control, Center for Infectious Diseases, California Department of Public Health, Richmond, California, United States of America; 2 Institute of Global Health, Global Health Sciences, University of California San Francisco, San Francisco, California, United States of America; University of California, San Francisco, United States of America

## Abstract

**Introduction:**

Tuberculosis (TB) requires at least six months of multidrug treatment and necessitates monitoring for response to treatment. Historically, public health departments (HDs) have cared for most TB patients in the United States. The Affordable Care Act (ACA) provides coverage for uninsured persons and may increase the proportion of TB patients cared for by private medical providers and other providers outside HDs (PMPs). We sought to determine whether there were differences in care provided by HDs and PMPs to inform public health planning under the ACA.

**Methods:**

We conducted a retrospective, cross-sectional analysis of California TB registry data. We included adult TB patients with culture-positive, pulmonary TB reported in California during 2007–2011. We examined trends, described case characteristics, and created multivariate models measuring two standards of TB care in PMP- and HD-managed patients: documented culture conversion within 60 days, and use of directly observed therapy (DOT).

**Results:**

The proportion of PMP-managed TB patients increased during 2007–2011 (p = 0.002). On univariable analysis (N = 4,606), older age, white, black or Asian/Pacific Islander race, and birth in the United States were significantly associated with PMP care (p<0.05). Younger age, Hispanic ethnicity, homelessness, drug or alcohol use, and cavitary and/or smear-positive TB disease, were associated with HD care. Multivariable analysis showed PMP care was associated with lack of documented culture conversion (adjusted relative risk [aRR] = 1.37, confidence interval [CI] 1.25–1.51) and lack of DOT (aRR = 8.56, CI 6.59–11.1).

**Conclusion:**

While HDs cared for TB cases with more social and clinical complexities, patients under PMP care were less likely to receive DOT and have documented culture conversion. This indicates a need for close collaboration between PMPs and HDs to ensure that optimal care is provided to all TB patients and TB transmission is halted. Strategies to enhance collaboration between HDs and PMPs should be included in ACA implementation.

## Introduction

Despite a decline in tuberculosis (TB) in the United States (U.S.) in the past two decades, TB remains a significant public health problem and is a challenging, resource-intensive disease to diagnose and treat. Treatment of active disease requires at least six months of a multidrug regimen and necessitates systematic monitoring for side effects and response to treatment. Because most TB patients have historically been managed by publicly funded local and state TB programs, [1] these programs have substantial expertise to successfully detect and treat TB disease in the U.S. However, the private sector plays an increasingly important role in diagnosing and treating TB. [2] As TB cases continue to decline in the U.S., [3] community health care providers may not see enough cases to build or maintain expertise in managing cases of TB. Regardless of the source of direct patient care, public health programs are responsible for oversight of TB patient treatment, to ensure that transmission is prevented. This need to protect the public from TB makes public-private collaboration crucial for effective management of TB. [2], [4],[5]

Effective management of TB should ensure timely conversion of sputum cultures to negative and prevent acquired drug resistance (ensure adherence to treatment). [6]–[8] Documenting prompt culture conversion also allows for the use of short-course TB therapy. [9] The practice of directly observed therapy (DOT) does not simply ensure treatment adherence, but also facilitates overall monitoring of treatment efficacy and provides patient support through structured contact with the health care system. [4], [9]

The Patient Protection and Affordable Care Act (ACA) [10] expands opportunities for patients to obtain health insurance and may increase health care provision in the private sector. In order to understand the potential impact of a shift in TB care from public TB programs to the private sector, we examined trends in providers caring for California TB patients over time, and examined differences in demographic and clinical characteristics of these two patient populations. We also sought to determine whether differences exist between care practices, including documenting that a patient has converted sputum cultures to negative and providing DOT to prevent acquisition of drug resistance.

## Materials and Methods

### Ethics statement

The California Department of Public Health (CDPH) routinely collects surveillance data, performs analyses and monitors trends for public health purposes. This analysis was determined to be a non-research public health analysis, and not subject to human subjects review. [11] All patient data were anonymized and de-identified prior to analysis.

### Analytic design

We used TB surveillance data in a retrospective, cross-sectional analysis to model the relationships between the provider type for TB care – within the public health department or outside the health department (e.g. private and other providers) – and two measures of optimal TB management: documenting culture conversion to negative, and ensuring treatment adherence through DOT.

### Data sources

TB surveillance data were captured through mandatory reporting by public health departments (HDs) of all TB cases to CDPH, using a standard report form containing demographic, clinical, and management information, including the type of clinical provider that managed the TB care. [12], [13] On the TB reporting form, a case was classified as “Health Department,” “Private/Other,” or “Both.” “Health Department” refers to patient care in a clinic directly managed by the public health department; for the vast majority of TB patients under HD care, this was a clinic devoted solely to TB diagnosis and treatment. “Private/Other” (hereafter private medical provider, or PMP) designates any other type of provider outside the public health department, including health maintenance organizations (HMOs), and county hospitals and clinics not directly managed by the HD. “Both” means that the patient received both HD and PMP care. Human immunodeficiency virus (HIV) infection status was determined by matching TB case records with the CDPH HIV/acquired immune deficiency syndrome registry.

### Inclusion criteria

**Figure 1:**
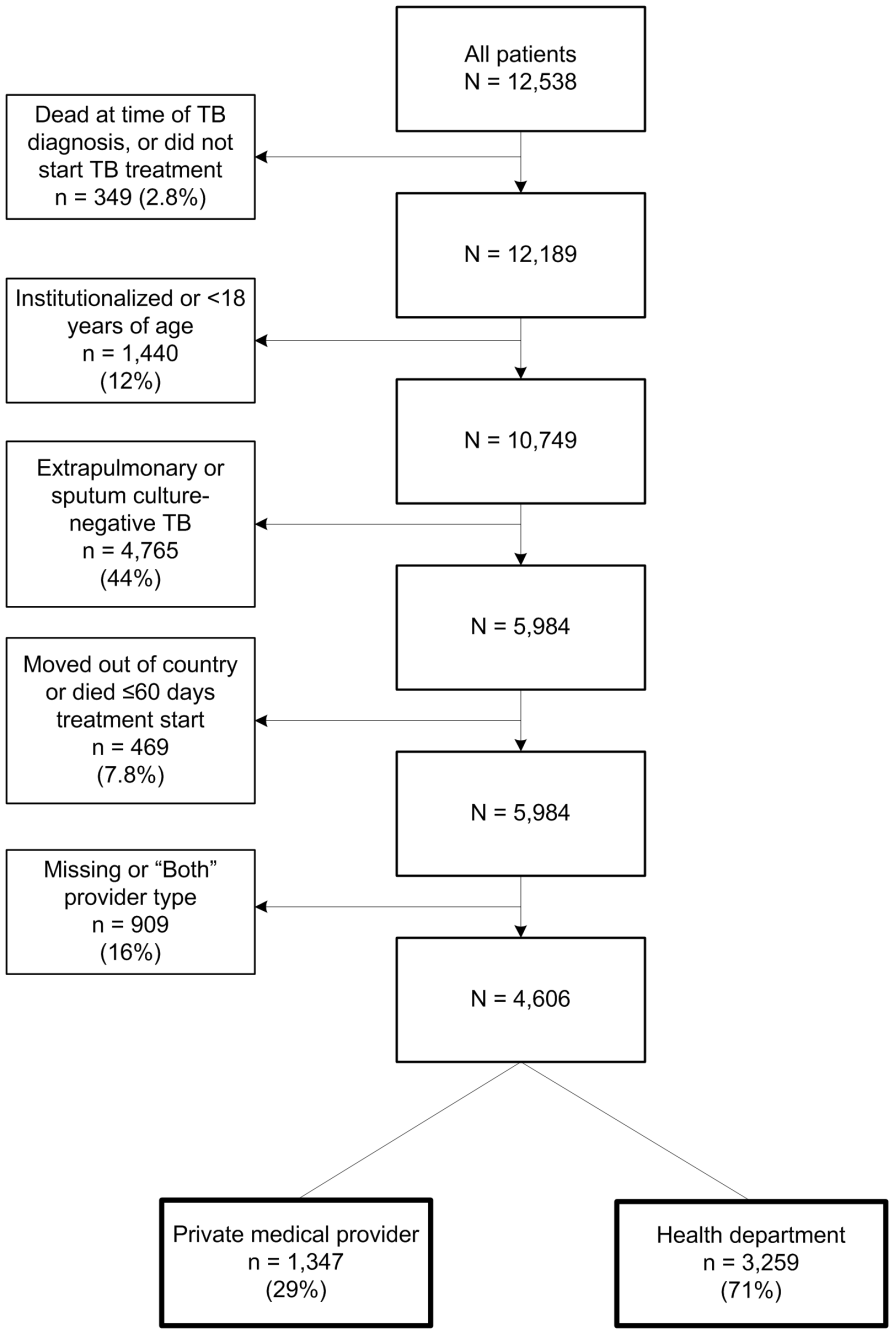
Analytic cohort, 2007–2011. TB  =  tuberculosis.

For analysis of TB management, TB cases reported to CDPH during 2007–2011 among persons 18 years and older with a sputum culture positive for *M. tuberculosis* and no extrapulmonary disease were selected (Figure 1). The study population inclusion criteria were designed to create a cohort of cases for which TB case management standards are best defined: culture-positive pulmonary TB in adults. [9] Patients who did not start TB treatment, died within 60 days of starting treatment, moved out of the U.S. during treatment, or were diagnosed in an institutional setting, i.e., a correctional or long-term care facility, were excluded. Because exclusive management of patients by PMPs or HDs are the most clearly defined and consistently reported provider types across California, [14] we limited the analysis to PMP and HD provider types, and excluded those designated as Both.

### Measures of optimal TB management

We examined two measures of TB management because of their importance for protecting the public's health: evidence of converting sputum culture from positive to negative during the initial treatment phase, and use of DOT. Calculations for these measures were based on indicators routinely used by CDPH [15] and the Centers for Disease Control and Prevention (CDC) [16] to monitor performance of TB control programs.

Culture conversion was defined as a report of a documented sputum culture-negative specimen which was collected within 60 days of the patient's treatment start date. We selected 60 days as the standard window used in clinical guidelines to assess treatment response and need for extension of therapy. [9] A patient was determined to have received DOT if therapy was reported to have been either totally directly observed, or both directly observed and self-administered.

### Data analysis

We examined trends, stratified by type of provider for TB cases, using Joinpoint version 4.0.4. [17] We fit the regression lines to the counts of PMP and HD patients during 1993–2011, identified join points that best fit the data using a Monte Carlo permutation test, and compared the trends for TB patients managed by PMPs compared to HDs, using a permutation test for parallelism. [18]

For all other calculations, we used SAS version 9.3. [19] We tested trends for statistical significance with the Cochrane-Armitage test for trend, for the study cohort and also for a cohort that included patients reported as managed by both PMPs and HDs. We compared PMP-managed patients to HD-managed patients for a range of clinical and demographic factors, and tested associations using the Mantel-Haenszel chi square test, or Fisher's exact test where any expected cell count was less than five. We assessed correlation with phi coefficient less than 0.30 for multivariable models. We combined race and ethnicity into mutually exclusive categories, where Hispanic ethnicity was coded as Hispanic, regardless of race; and white, black, and Asian race categories each excluded persons of Hispanic ethnicity. In the multivariable models, we used birth in the U.S. instead of race/ethnicity categories due to correlation of these two variables. Other correlated variables were combined into categories: smear-positive and cavitary TB disease, and homelessness and substance use (alcohol, injecting and non-injecting). We modeled the association of provider type with the two measures of TB management in univariable and multivariable analyses. We calculated crude and adjusted relative risk using modified Poisson regression with robust error variance. [20], [21] Covariables associated with both provider type and measures of TB case management, and factors cited in the literature as possible confounders, were included *a priori* in each multivariable model. We compared models with additional covariables in different combinations using the QIC statistic (quasi-likelihood under the independence model criterion). [22] We selected the model with the lowest QIC as the final model. To assess the impact of excluding patients reported with a provider type of Both (PMP and HD), we analyzed a cohort that included patients managed by Both, and with all other study exclusions. We constructed multivariate models using this cohort in two ways: 1) PMP+Both vs. HD, and 2) PMP vs. HD+Both. We conducted a sensitivity analysis of the relationship between PMP care, DOT, and death during therapy, by excluding patients who died at varying times, to assess the robustness of the 60-day cut point for excluding deaths. We created two separate models to investigate documented culture conversion: documented culture conversion in greater than 60 days after starting treatment, and lack of documented culture conversion ever, to assess whether PMP care was associated with never having a culture conversion documented, or with culture conversion documented after 60 days of treatment. Because California guidelines prioritize key patient groups for DOT when resources do not allow for universal DOT, [23] such as homeless or those with drug-resistant disease, we conducted a subanalysis of predictors of DOT with a cohort restricted to patients with at least one indication for DOT.

## Results

Among the 12,538 TB patients reported in California during 2007–2011, 4,606 were included in the analysis (Figure 1). While most patients were managed by the HD (3,259 or 71%), 29% (1,347) were privately managed.

**Figure 2:**
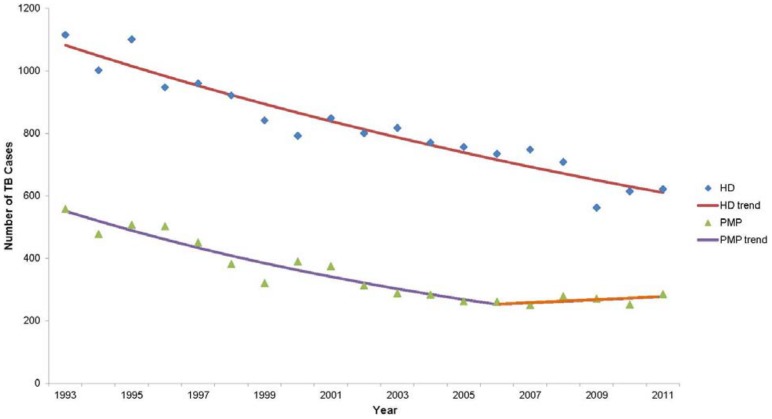
Tuberculosis case count trend, by provider type, California 1993–2011. HD  =  health department, PMP  =  private medical provider. Annual percent change (APC): HD 1993–2011 = −3.13, PMP 1993–2006 = −5.83, PMP 2006–2011 = +1.85.

The number of TB patients reported in California steadily declined during 1993–2011, [24] and TB cases meeting our study population criteria also declined during this time. Figure 2 shows the differences in TB incidence trends between public and private sectors. During 1993–2006, the incidence of HD TB patients declined 3.1% per year (95% confidence interval [CI] −3.6, −2.7); whereas PMP patients declined 5.8% per year (CI −7.0, −4.7). During 2006–2011, however, the decline in PMP-managed TB cases leveled off (annual percent change [APC] = 1.9, CI −4.2, 8.3), whereas HD-managed patients continued to decline at the previous rate. This difference in the rate of incidence decline was statistically significant (p = 0.001).

In our analytic cohort, the proportion of cases with PMP care increased, rising from 25% in 2007 to 32% in 2011 (p = 0.002). In the modified study cohort that included patients with a provider type of Both, the proportion of patients categorized as Both declined from 18% in 2007 to 8.8% in 2011 (p<0.001).

### Univariable analysis

Table 1 shows the characteristics of cases managed by PMPs compared with cases managed by HDs. Older patients, those of white, black or Asian/Pacific Islander race, or born in the U.S., were all significantly more likely to have PMP care. Patients cared for by HDs were more likely to be young, Hispanic, homeless, excess alcohol users, non-intravenous drug users, or have cavitary and/or smear-positive TB disease. No patients reported stopping treatment or having treatment extension beyond 12 months due to adverse reactions to TB medication. Documented culture conversion and DOT were observed less frequently among patients managed by PMPs.

**Table 1:** Characteristics of TB Patients by Provider Type, Reported in California, 2007–2011 (n = 4606).

	All patients^a^	PMP (n = 1347)	HD (n = 3259)	PMP vs. HD
Characteristic	n (col %)	n (col %)	n (col %)	P value^b^
Male	2893 (63)	819 (61)	2074 (64)	0.070
18–24 years old	456 (9.9)	100 (7.4)	356 (11)	<0.001
25–44 years old	1421 (31)	366 (27)	1055 (32)	<0.001
45–64 years old	1611 (35)	423 (31)	1188 (36)	0.001
Age ≥65 years old	1118 (24)	458 (34)	660 (20)	<0.001
White	369 (8.0)	147 (11)	222 (6.8)	<0.001
Black	283 (6.2)	110 (8.2)	173 (5.3)	<0.001
Hispanic	1586 (34)	293 (22)	1293 (37)	<0.001
Asian/Pacific Islander	2359 (51)	797 (59)	1562 (48)	<0.001
American Indian/Alaskan Native	8 (0.2)	0 (0)	8 (100)	0.114
Born in United States	792 (17)	297 (22)	495 (15)	<0.001
Moved ever during treatment	234 (5.1)	72 (5.4)	162 (5.0)	0.599
Homeless	273 (5.9)	53 (3.9)	220 (6.8)	<0.001
Excessive alcohol use	431 (9.4)	98 (7.3)	333 (10)	0.002
Intravenous drug use	40 (0.9)	11 (0.8)	29 (0.9)	0.804
Nonintravenous drug use	250 (5.4)	51 (3.8)	199 (6.2)	0.002
Homelessness, alcohol or drug use	682 (15)	143 (11)	539 (17)	<0.001
History of TB disease	286 (6.3)	89 (6.7)	197 (6.1)	0.448
HIV-positive^c^	143 (3.1)	47 (3.4)	96 (3.0)	0.333
Cavitary and/or smear-positive TB disease	3183 (70)	881 (66)	2302 (71)	<0.001
Resistance to any first-line TB drug	651 (14)	183 (14)	468 (14)	0.506
No documented culture conversion ≤60 days	1469 (32)	533 (40)	936 (29)	<0.001
No directly observed therapy	342 (7.4)	270 (20)	72 (2.2)	<0.001

a alive at diagnosis and starting treatment, pulmonary only, culture-positive TB, age ≥18 years old; excluding missing or Both provider type, moved out of United States, deaths ≤60 days after treatment start.

b for Mantel-Haenszel chi-square, or Fisher's Exact Test where any expected cell count is <5.

c vs. HIV-negative or unknown status.

TB = tuberculosis, PMP = private medical provider, HD = health department, HIV = human immunodeficiency virus.

### Multivariable analysis

A total of 4,604 (99.9%) patients in the analytic cohort had complete information on culture conversion. The absence of documented culture conversion within 60 days of treatment start was significantly associated with privately managed care in multivariable analysis (adjusted relative risk [aRR] 1.37, CI 1.25–1.51). This model adjusted for ten covariables including age, cavitary and/or smear-positive TB disease, and drug resistance. In addition to private medical care, factors predicting lack of documented culture conversion in the adjusted model included: cavitary and/or smear-positive TB disease, moving during treatment, no DOT, birth in the U.S., male sex, and any homelessness or drug use (Table 2). Privately managed care was a significant predictor of both never having culture conversion documented (aRR 3.34, CI 2.54–4.36) and of delayed culture conversion, documented later than 60 days (aRR 1.24, CI 1.12–1.39).

**Table 2:** No Documented Culture Conversion within 60 Days of TB Treatment Start, TB Cases Reported in California, 2007–2011 (n = 4604).

	All patients^a^	No CC (n = 1469)	CC (n = 3135)	Univariable		Multivariable	
Characteristic	(col %)	(col %)	(col %)	RR	CI	aRR	CI
Private provider/other	1346 (29)	533 (36)	813 (26)	1.38	1.27–1.50	1.37	1.25–1.51
Male	2893 (63)	994 (68)	1899 (61)	1.24	1.13–1.36	1.19	1.08–1.31
Age ≥65 years old	1118 (24)	341 (23)	777 (25)	0.94	0.85–1.04	0.95	0.86–1.06
Born in United States	792 (17)	313 (21)	479 (15)	1.31	1.19–1.44	1.18	1.06–1.31
Moved ever during treatment	234 (5.1)	94 (6.4)	140 (4.4)	1.28	1.09–1.50	1.23	1.04–1.46
Homelessness, alcohol or drug use	692 (15)	269 (18)	413 (13)	1.29	1.16–1.43	1.16	1.04–1.30
History of TB disease	286 (6.3)	97 (6.7)	189 (6.1)	1.07	0.91–1.27	1.09	0.92–1.29
HIV-positive^b^	143 (3.1)	41 (2.8)	102 (3.3)	0.90	0.69–1.16	0.82	0.63–1.08
Cavitary and/or smear-positive TB	3181 (70)	1168 (80)	2013 (65)	1.73	1.54–1.93	1.80	1.61–2.02
Resistance to any first-line TB drug	651 (14)	182 (13)	469 (15)	0.86	0.76–0.98	0.91	0.80–1.04
No directly observed therapy	342 (7.4)	138 (9.4)	204 (6.5)	1.30	1.13–1.48	1.24	1.07–1.45

a alive at diagnosis and starting treatment, pulmonary only, culture-positive TB, age ≥18 years old; excluding missing or Both provider type, moved out of United States, deaths ≤60 days after treatment start.

b vs. HIV-negative or unknown status.

TB = tuberculosis, CC = culture conversion, RR = relative risk, CI = 95% confidence interval, aRR = adjusted relative risk, HIV = human immunodeficiency virus.

Nearly all patients (99.5%) had complete information on therapy administration method (DOT or self-administered therapy [SAT]). In the adjusted model, patients under private care were significantly more likely to have received SAT during the course of TB treatment, and PMP care was the strongest predictor of SAT among all covariables (aRR 8.56, CI 6.59–11.1). Lack of documented culture conversion within 60 days was also associated with SAT. Patient characteristics associated with receipt of DOT included homelessness or drug use, cavitary and/or smear-positive TB, a history of TB disease, resistance to any first-line drug used to treat TB, and age 65 years old or greater (Table 3). Similar results were found when excluding patients who died during therapy at 30 or 90 days. Among patients who received DOT, 85% received at least 21 weeks of DOT, and 94% received at least 10 weeks of DOT.

**Table 3:** No Directly Observed Therapy throughout TB Treatment, TB Cases Reported in California, 2007–2011 (n = 4585).

	All patients^a^	No DOT (n = 342)	DOT (n = 4226)	Univariable		Multivariable	
Characteristic	(col %)	(col %)	(col %)	RR	CI	aRR	CI
Private provider/other	1335 (29)	270 (79)	1065 (25)	9.13	7.10–11.7	8.56	6.59–11.1
Male	2880 (63)	196 (57)	2684 (63)	0.79	0.64–0.98	NA	NA
Age ≥65 years old	1108 (24)	97 (28)	1011 (24)	1.24	0.99–1.56	0.79	0.64–0.99
Born in United States	791 (17)	68 (20)	723 (17)	1.20	0.93–1.54	NA	NA
Moved ever during treatment	228 (5.0)	23 (6.7)	205 (4.8)	1.38	0.92–2.06	NA	NA
Homelessness, alcohol or drug use	682 (15)	16 (4.7)	666 (16)	0.28	0.17–0.46	0.39	0.24–0.66
History of TB disease	284 (6.3)	14 (4.1)	270 (6.4)	0.64	0.39–1.09	0.53	0.30–0.94
HIV-positive^b^	143 (3.1)	5 (1.4)	138 (3.3)	0.46	0.19–1.10	0.51	0.21–1.22
Cavitary and/or smear-positive TB disease	3168 (70)	150 (45)	3018 (72)	0.35	0.29–0.43	0.41	0.33–0.49
Resistance to any first-line TB drug	648 (14)	31 (9.2)	617 (15)	0.61	0.43–0.88	0.64	0.46–0.91
No documented culture conversion ≤60 days	1459 (32)	138 (40)	1321 (31)	1.44	1.18–1.78	1.23	1.01–1.49

a alive at diagnosis and starting treatment, pulmonary only, culture-positive TB, age ≥18 years old; excluding missing or Both provider type, moved out of United States, deaths ≤60 days after treatment start.

b vs. HIV-negative or unknown status.

TB = tuberculosis, DOT = directly observed therapy, RR = relative risk, CI = 95% confidence interval, aRR = adjusted relative risk, NA = not in final multivariate model, HIV = human immunodeficiency virus.

In a subanalysis, limited to patient groups prioritized for DOT under California guidelines, PMP care also had the greatest association with lack of DOT (aRR 6.46, CI 4.61–9.06). Patient groups for whom DOT is a priority in California include the homeless, drug or alcohol users, HIV infected, and patients with drug-resistant TB, smear-positive TB, delayed culture conversion, or prior history of TB.

Multivariable models using the modified study cohort including Both HD and PMP patients showed similar results for each study outcome, with no change in the direction nor significance of association for any covariable.

## Discussion

We found that during 2007–2011, before implementation of the ACA, an increasing proportion of TB cases in California were exclusively managed by PMPs. We also found that patients managed by PMPs were less likely to have received optimal management of their pulmonary TB even after adjusting for possible confounding clinical and social factors. These findings may have implications for TB care under the ACA, when a more dramatic shift of TB care to the private sector is expected. If PMP patients are less likely to receive optimal management, then shifting toward PMP care without a concomitant increase in HD partnership with PMPs could be a threat to TB control.

Our findings are consistent with evidence showing that patients in public health TB clinics are more likely to receive care recommended in TB control guidelines. [25]–[31] Similar findings have been published in which clinicians and clinics with the most experience and volume provide care resulting in better outcomes to patients for other diagnoses such as HIV [32] and *Staphylococcus aureus* bacteremia. [33]–[35] The results also highlight the concept that public health TB clinics and programs warrant consideration as expert, specialty referral clinics, and not simply government sector safety net providers. In fact, specialty TB care has been established across the U.S. since the early 1900s, and has been intentionally centered in public health TB clinics since the 1960s. [1] These TB specialty clinics provide patient-centered clinical care, case management, and monitoring for persons with complex health issues over months of treatment. However, TB control programs are charged with “ensuring the quality and completeness” [4] of care for all patients and these findings also point out that there should be close collaboration between PMPs and TB control programs to ensure that patients managed exclusively by PMPs receive the same optimal care that patients managed exclusively by the HD do.

Further highlighting the important role of HD TB care, we found that HDs care for a disproportionate burden of complex patients, such as homeless or drug users, who may be more difficult to treat. HDs also will remain critical “safety net” providers for populations uncovered by ACA, such as undocumented immigrants, who comprise an estimated 23% of persons with active TB disease in the United States. [36] Regardless of where TB patients access care, any lack of optimal care that results in increased transmission would increase the risk of TB in the community, and require heightened response from HDs in California, because they will retain responsibility for outbreak management and contact investigations. Shifting patients from HD to PMP care may therefore increase the need for HD resources, rather than lessen it. Studies have shown that increased funding is associated with better public health performance, [37] specifically for infectious disease morbidity. [38]

Our analysis assessed two aspects of clinical TB care that are modifiable and important. Documenting sputum culture conversion to negative during the initiation phase of treatment is a key clinical measure of treatment efficacy, and allows determination of appropriate length of therapy. [9] Directly observed therapy is a strategy widely used throughout the world to ensure treatment adherence. [9], [39] Providing DOT has been shown to reduce rates of acquired drug resistance. [6]

There are many reasons PMPs may not provide DOT or collect sputum cultures for TB patients, including 1) financial resources, 2) a lack of knowledge of how to provide DOT and sputum induction, and 3) lack of understanding of the value of DOT and need to document sputum culture results during treatment. There is also a tradition of HDs providing DOT for patients. [1] However, HDs with limited resources may not be able to provide DOT or sputum induction to PMP patients, or may have policies preventing this. In California, sputum induction and HD-provided DOT are reimbursable procedures under Medicaid, [40] but capacity and agreements to bill private insurance for services provided by the health department are not in place in many HDs. Encouragement under the ACA for accountable care organizations to include HD TB specialists in their group of providers might promote HD-PMP collaboration in a way that is reimbursable for the HD. Ensuring that HDs have policies and procedures in place to facilitate access to sputum induction and DOT for PMP patients is an important first step.

With an anticipated increase in private care, and a history of uneven assurance of private TB care by HDs, how will TB care be strengthened for privately-managed patients? Despite barriers, improvement is possible and has been demonstrated by California TB control programs that systematically and routinely review PMP-managed patients during case conferences. [41] Health departments have reported success in working with PMPs on TB prevention when HDs have administrative support, have a toolkit of educational information, protocols and forms, and work to develop relationships with PMPs in the community. Alerts generated by electronic health records that could trigger PMP action such as collecting sputum or notifying the HD may also contribute to success. These lessons from TB prevention could be applied to HD-PMP partnership for active TB treatment as well. [42] These interventions can be replicated in HDs undergoing fiscal cuts. The HD staff time needed to put oversight, tracking and collaborative measures in place can save time downstream as follow-up and patient care issues are averted.

Two additional points deserve comment. First, the measures of patient care analyzed in this paper are interrelated. Lack of documented culture conversion and not receiving DOT were each far more likely among PMP patients, and each also predicted the other in multivariable models. This was expected because DOT and monitoring culture conversion are case management activities overseen by the HD for both privately and publicly managed patients. These activities are likely to occur together as part of routine monitoring. We adjusted for each practice in the multivariable models to account for confounding. Second, several groups of patients were found to be more likely to receive DOT, including those with previous TB, cavitary or smear-positive TB, drug resistance, or social factors complicating treatment. This was not surprising because in California, patients with these factors are prioritized for DOT. In both the main analysis, and the subanalysis which included only patients with at least one indication for DOT in California, PMP care remained the strongest independent predictor of not receiving DOT.

Our analysis is subject to some limitations. First, our exclusion criteria were constructed to focus on adults with culture-positive, pulmonary TB, and as a result may not be generalizable to extrapulmonary, pediatric, or culture-negative TB cases. However, the relationship between PMPs and the measures of interest held even among excluded groups of cases where sample sizes were sufficient and analysis was possible, e.g., we could not assess culture conversion among culture-negative cases. Second, information on provider type in TB surveillance data may be subject to misclassification because HDs may not apply a uniform definition. [14] Patients reported as managed by Both HD and PMP may be co-managed, or sequentially managed, depending on local policies and procedures or individual situation; these distinctions would affect the interpretation of results for this category of patients. However, we restricted our analytic cohort to cases managed solely by either private or public health providers, types for which the definitions are clearest. We also showed that the proportion of patients reported as Both HD and PMP was small and declining, and that grouping these patients with either HD or PMP patients did not affect the overall results. Third, we were unable to distinguish between types of private medical provider settings (e.g., HMOs, private medical practices, non-profit clinics, and private or non-HD public hospitals). Aggregating disparate provider groups in our analysis may have masked important findings specific to one type of private provider. Stratified analysis by these private provider types, or even by specific large provider groups, would be an important next step in determining tailored interventions. Last, the TB report form was unable to distinguish whether a recorded delay in culture conversion was due to a delay in sputum collection, or persistent culture-positive sputum. Therefore, it may be that PMPs are equally able to render patients culture negative but PMP patients are less likely to have this documented in the public health record in a consistent and timely way. However, PMP care predicted both delayed culture conversion and never documented conversion in our analysis. Regardless of the reason for delayed culture conversion, our findings show the need for increasing PMP education and collaboration between HDs and PMPs.

In conclusion, our findings point to an opportunity for improvement in clinical care provided to TB patients managed exclusively in the private sector. Implementation of the ACA should take into account the TB expertise found in public sector TB programs, reinforce the role of public health departments to ensure optimal treatment of persons with TB disease, and make resources for key TB management practices available and accessible to all patients, regardless of where care is received.
